# Studies on plasmids of multidrug resistant uropathogens isolated from patients with urinary tract infection in a tertiary hospital in Calabar, Nigeria

**DOI:** 10.4314/ahs.v23i1.81

**Published:** 2023-03

**Authors:** Juliet Nnedinma Iheanacho, Sylvester Peter Antai

**Affiliations:** Department of Microbiology, Faculty of Biological Sciences, University of Calabar, Nigeria

**Keywords:** Resistance, plasmid, UTI, uropathogens, curing

## Abstract

**Background:**

Urinary tract Infections caused by multidrug resistant uropathogens have become a significant global public health problem with Nigeria being no exception.

**Objective:**

This study is aimed at profiling and curing the plasmids of selected multidrug resistant uropathogens isolated from patients with urinary tract infection in a tertiary hospital in Calabar, Nigeria.

**Methodology:**

Isolates were obtained from urine samples of patients using standard microbiological techniques. Multidrug resistant bacterial isolates were then selected for plasmid DNA analysis and curing.

**Results:**

The study revealed that *E. coli, K. pneumoniae, P. aeruginosa* and *Proteus mirabilis* were resistant to the antibiotics tested. The extracted plasmid DNA showed the presence of TEM, SHV and CTX-M genes in the isolates with sizes of 400-600bp, 300bp and 500-800bp, respectively. All isolates possessed the SHV genes while few had TEM and CTX-M genes. Cells were subjected to curing and plasmid curing was achieved at 200-300µl of ethidium bromide.

**Conclusion:**

The reduction in percentage resistance due to plasmid curing observed in this study suggests that the resistance of the isolates to antibiotics were plasmid-mediated. Antibiogram and monitoring of plasmid mediated resistance are necessary for proper management of urinary tract infections.

## Introduction

Urinary tract infections are common bacterial infections. The injudicious use of antibiotics have resulted in increased resistance of microorganisms to commonly used antibiotics^6^. The global evolution in antibiotic resistance has necessitated research into the prevalence of antibiotic resistant bacteria and possible mechanisms of resistance in different environments^3^. Multidrug resistance (MDR) in bacteria is often the result of acquisition of mobile genetic elements that contain multiple resistance genes.^8^

One of the mobile genetic elements that facilitates the spread of antibiotic resistance in bacteria are plasmids. They contain about 2% of a cell's genetic information and they give selective advantage such as heavy metal resistance or resistance to antibiotics^19^. The studies carried out to ascertain the role of plasmids in antibiotic resistance are useful in determining the characteristics of plasmids in bacterial cells^11^. This study is therefore aimed at profiling and curing the plasmids of selected multidrug resistant bacterial species isolated from patients with urinary tract infection in a tertiary hospital in Calabar Metropolis.

## Methods

### Sample collection

Two hundred clean voided mid-stream urine samples were obtained from patients visiting a tertiary hospital in Calabar, Nigeria. Samples were collected in sterile universal bottles and analyzed following standard microbiological techniques.

### Sensitivity Testing

Bacteria isolates were subjected to antibiotics sensitivity test using the Kirby Bauer disc diffusion as recommended by the Clinical Laboratory Standard Institute (CLSI, 2015). The antibiotics used were gentamicin (10µg), augmentin (30µg), ofloxacin (5µg), ciprofloxacin (5µg), nitrofurantoin (300µg), ceftazidime (30µg), cefuroxime (30µg) and ampicillin (10µg).

Bacteria isolates that were resistant to all the antibiotics tested were selected for plasmid analysis.

### Plasmid DNA Isolation

Multidrug-resistant bacteria isolates were grown overnight in Luria-Bertani (LB) broth at 37°C after which plasmid DNA was extracted from lyzed isolates using the ZYPPYTM Plasmid Miniprep Kit, (Inqaba Biotech., South Africa) following alkaline lysis method described1.

### Amplification of plasmid DNA

Extracted plasmid DNA were amplified by standard PCR. Oligonucleotide primers of the TEM, SHV and CTX-M genes used were TEM: F-5′-TCCGCTCATGAGACAATAACC-3″, R-3′-ATAATACCGCACCACATAGCAG: SHV: F-5′-CGCCTGTGTATTATCTCCCT-3″, R-3″ CGAGTAGTCCACCACCAGATCCT-5′, CTX-M: F-5′-CGCTTTGCGATGTGCAG-3″, R-3′-ACCGCGATATCGTTGGT-5′ on a ABI 9700 Applied Biosystems thermal cycler at a final volume of 50 microliters for 35 cycles. Amplicons were resolved on a 1.5% agarose gel at 120V for 35 minutes and visualized on a UV transilluminator.

### Plasmid Curing and Re-evaluation of Antibiotic Susceptibility profile of bacterial isolates

Plasmid curing was carried out following methods described by Raghada^13^. Briefly, 10ml of each bacterial culture inoculated into peptone water and incubated for 24hrs was introduced into a set of 18 test tubes, respectively. Ethidium bromide in various concentrations of 0, 20, 50, 100, 150, 200, 250, 300, 350, 400, 450, 500, 550, 600,650, 700, 750 and 800 µl were then introduced accordingly into the test tubes and incubated for 24 hrs at 37°C to determine the sub-lethal concentration of ethidium bromide. After incubation, 1ml aliquot from each test tube was inoculated onto nutrient agar plates and incubated. Cured colonies were selected and inoculated onto freshly prepared Muller Hinton agar plates. Then, antibiotic discs of prior resistance were aseptically introduced into the plates and incubated for 24 hrs at 37°C after which plates were examined for susceptibility.

## Results

### Antibiotic Resistance Pattern of Selected Bacteria for Plasmid Analysis

Selected bacteria isolates: Escherichia coli, Klebsiella pneumoniae, *Pseudomonas aeruginosa* and Proteus species were all resistant to eight (8) antibotics, respectively as shown in Table 1.

**Table 1:** Antibiotic resistance pattern of selected bacterial isolates for plasmid analysis

Bacterial isolates	Antibiotic resistance
*Escherichia coli* (3)	CPR, OFL, AUG, NIT, AMP, CAZ, CRX, GEN
*Klebsiella pneumoniae* (3)	OFL, AUG, NIT, CPR, AMP, CRX, GEN, CAZ
*Pseudomonas aeruginosa* (2)	AUG, NIT, AMP, CAZ, CRX, GEN, CPR, OFL
*Proteus* spp. (2)	CPR, OFL, AUG, NIT, AMP, CAZ, CRX, GEN

**KEY:** OFL-Ofloxacin, CPR-Ciprofloxacin, AUG-Augmentin, GEN- Gentamycin, AMP-Ampicillin, CAZ-Ceftazidime, NIT- Nitrofurantoin, CRX- cefuroxime

### Plasmid analysis of resistant isolates

**Plate 1:**
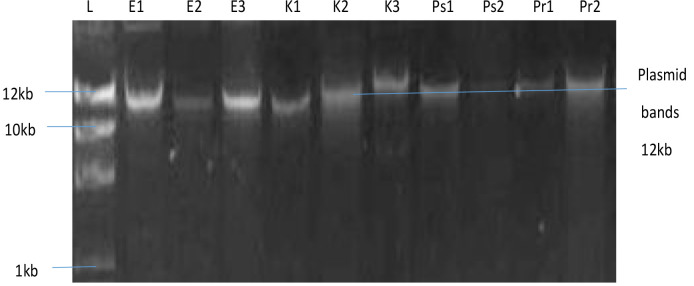
Agarose gel electrophoresis of the plasmid of the bacterial isolates. Lane L represents a 1kb molecular ladder. **Key:** E-Escherichia coli, K-Klebsiella pneumoniae, Ps-Pseudomonas aeruginosa, Pr-Proteus mirabilis

The plasmid profile of bacterial isolates is presented on plate 1. All ten isolates possessed plasmids of 12kb (1kb molecular ladder).

### Distribution of TEM, SHV and CTX-M Genes among Test Isolates

**Plate 2:**
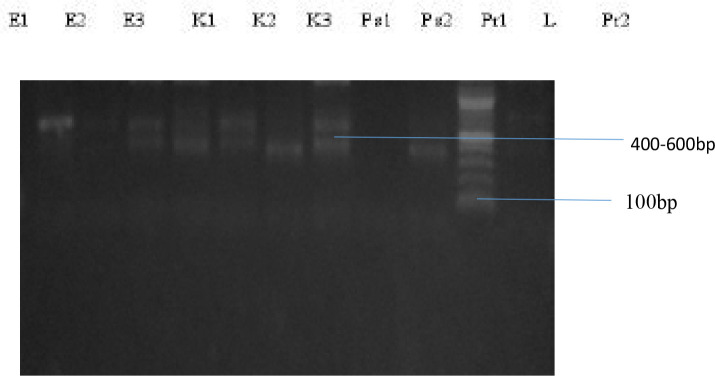
Agarose Gel Electrophoresis showing TEM gene. Lane L represent the molecular ladder (100bp) Key: *E-Escherichia coli, K-Klebsiella pneumoniae, Ps-Pseudomonas aeruginosa, Pr-Proteus mirabilis*

**Plate 3:**
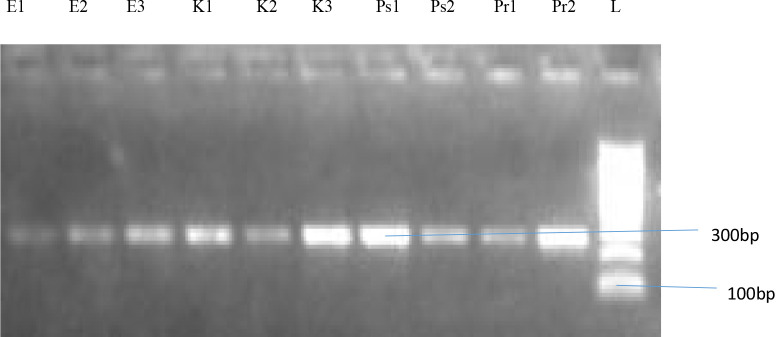
Agarose Gel Electrophoresis showing SHV gene of 300bp. Lane L represent a molecular ladder (100bp). Key: *E-Escherichia coli, K-Klebsiella pneumoniae, Ps-Pseudomonas aeruginosa, Pr-Proteus spp*.

**Plate 4:**
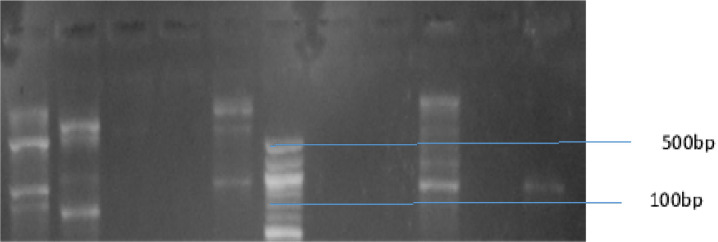
Agarose Gel Electrophoresis showing CTX-M gene. Lane 1-10 represents the isolates were 1, 2, 5, 8 and 10 were positive while 3, 4, 6, 7 and 9 showed no amplification. Lane L represent the molecular ladder (100bp) Key: *E-Escherichia coli, K-Klebsiella pneumoniae, Ps-Pseudomonas aeruginosa, Pr-Proteus spp.*

**Figure 1:**
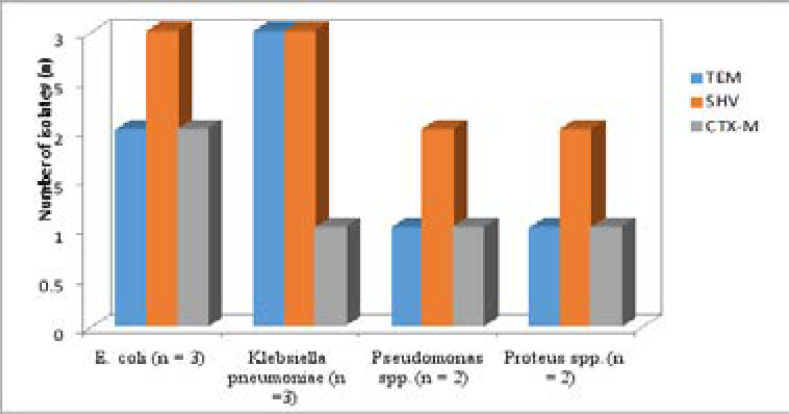
Distribution of TEM, SHV and CTX-M genes among the bacterial isolates

**Figure 2:**
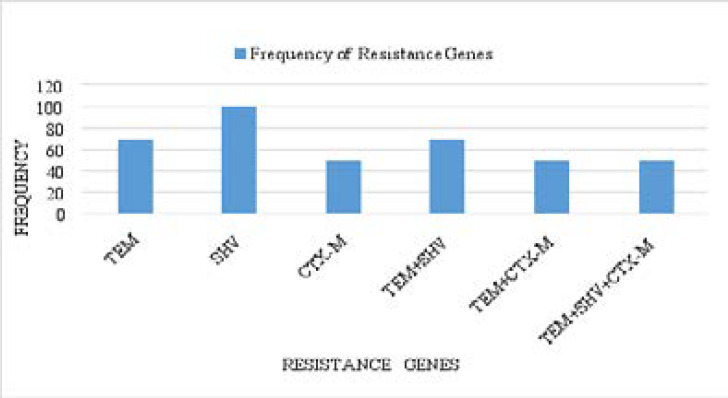
The frequency of occurrence of TEM, SHV and CTX-M genes among bacterial isolates

A representation of the bands corresponding to the various genes is shown on Plates 2, 3 and 4, respectively. The entire test isolates employed in this analysis possessed the SHV genes 10(100%) while only 2(66.7%) of *Escherichia coli* strains possessed the TEM and CTX-M genes. All the three (3) strains of *Klebsiella pneumoniae* (100%) had the TEM genes while only 1(33.3%) possessed the CTX-M genes. Furthermore, of the two strains of *Pseudomonas aeruginosa* and *Proteus mirabilis* analysed, only 1(50%) possessed the TEM and CTX-M genes. The distribution of resistance genes among test isolates is shown in Fig. 1 while the frequency of occurrence of resistance genes among resistant isolates is shown in Fig. 2.

### Plasmid curing of bacterial isolates

All the bacterial isolates possessing resistant plasmids where subjected to treatment (curing) with ethidium bromide and were found to exhibit various degrees of growth. At 100µl – 200µl *Pseudomonas aeruginosa* and Proteus mirabilis showed moderate growth (+) while *E. coli* and *K. pneumoniae* showed slight growth (+). At 300µl, *Escherichia coli* and *Klebsiella pneumoniae* showed no growth (-) while *Pseudomonas aeruginosa* and *Proteus mirabilis* exhibited slight growth (+). At 400 – 800µl none of the isolates exhibited growth as presented in Table 3.

### Susceptibility Pattern of Cured Isolates to Antibiotics

The susceptibility pattern of cured isolates when subjected to antibiotics was determined. Interestingly, all the cured Escherichia coli, Klebsiella pneumoniae, Pseudomonas aeruginosa and Proteus mirabilis were susceptible to all the antibiotics they were previously resistant to.

## Discussion

Urinary tract infection (UTI) is responsible for frequent visits of in and outpatients to the hospital regularly. Despite the increase in the advancement in antimicrobial therapy UTI still remains a major cause of morbidity. The high rate of resistance to commonly used antibiotics observed in this study raises great concern over the available options to treat UTIs. The rise in the frequency and spectrum of antibiotic resistance in recent years is a major public health concern. The acquisition of resistance genes by horizontal transfer play a major role in the development of MDR strains of microorganisms^5^. One of the mobile genetic elements through which antibiotics resistance spreads in bacteria are plasmids^2^.

Plasmid profiling of the MDR uropathogens in this study revealed that the isolates contained R-plasmids with molecular weight of 12kb. The emergence of R-plasmids in this study could be ascribed to the indiscriminate use of antibiotics caused by over- the- counter availability of antibiotics^4^,^6^. The plasmid DNA of the isolates harboured the SHV, TEM and CTX-M genes with each isolate harbouring at least one type of resistant gene. All the three strains of *E. coli* harboured the SHV genes while only two possessed the CTX-M and TEM genes. The finding of the SHV in E. coli strains is in accordance with previous report of SHV mediated resistance in 28.6% of *E. coli* strains in Turkey^10^. In recent years, CTX-M gene has been implicated in extended spectrum ESBL production in *E. coli* which is usually plasmid mediated. The finding of CTX-M in these isolates is consistent with reports of Oteo et al.^12^ and Amaya et al.^7^ who reported that 30.8% and 26% of *E. coli* strains observed in their studies respectively possessed blaCTX-M enzymes which hydrolyse mainly cefotaxime but weakly active against Ceftazidime. The CTX-M enzymes are known as an increasingly serious public health concern worldwide and have been noted to be the cause of outbreaks^13^. Shacheraghi et al.^16^ reported the prevalence of TEM and SHV genes in E. coli to be 46.4% and 11.2% respectively. The three strains of *K. pneumoniae* which were subjected to plasmid analysis possessed SHV and TEM genes while only one possessed the CTX-M gene. Studies have shown that strains of *K. pneumoniae* constitutively express chromosomal class A blactamases including SHV, CTX and TEM and are wide spread globally^9^. The presence of these genes in the plasmid DNA of *K. pneumoniae* in this study suggests that the production of the SHV, TEM and CTX-M β-lactamases in these organisms is both chromosomal and plasmid encoded. These plasmids could have resulted from environmental interactions with other plasmid bearing members of the Enterobacteriaceae family probably due to the selection pressure from the continuous prescription and misuse of broad spectrum antibiotics^9^. As reported by El- Bouamri et al.^9^ ESBL (CTX-M) encoding genes serve as additional resistance determinants to aminoglycosides and fluoroquinolones since they are often co-transferred on the same plasmid.

The two strains of *P. aeruginosa* analysed in this study possessed the SHV genes while only one had the TEM and CTX-M genes. According to Picao et al.^14^ the presence of CTX-M gene in *P. aeruginosa* confirms the assertion that this gene is not exclusive to Enterobacteriaciae family.

The two Proteus species possessed the SHV gene while one isolate possessed the CTX-M and TEM genes respectively. This observation agrees with report of Aragon18 who confirmed that Proteus species lack intrinsic chromosomally encoded b-lactamase genes and as such, depend entirely on acquisition of various beta-lactamase genes to express a beta-lactamase mediated resistance phenotype. Furthermore, CTX-M β-lactamase has been shown to be responsible for the resistance of Proteus species to antibiotics^18^.

Bacterial isolates in this study exhibited varied pattern of growth at the various concentrations of ethidium bromide. Plasmid curing resulted in susceptibility of the bacterial isolates to all the antibiotics they were initially resistant to. Spengler et al.^17^ reported that curing agents like ethidium bromide act on plasmids either via inhibition of plasmid efflux pumps or inhibition of DNA gyrase responsible for plasmid DNA replication. The reduction in percentage resistance due to plasmid curing observed in this study suggests that the resistance of the isolates to antibiotics were plasmid-mediated.

## Conclusion

The presence of resistance plasmids harbouring beta-lactamase genes in clinical isolates could contribute to the dissemination of multidrug resistant bacteria thus posing a serious threat to public health. We therefore recommend antibiogram and monitoring of plasmid mediated resistance for proper management of UTI and also to avoid treatment failure.

## Figures and Tables

**Table 2:** Effect of Ethidium Bromide on the growth of selected resistant bacterial isolates

Ethidium bromide (mg/µl)	*Escherichia* *coli.* (3)	*Klebsiella spp.* (3)	*Pseudomonas* *spp.* (2)	*Proteus spp.* *(2)*
0	+++	+++	+++	+++
20	+++	+++	+++	+++
50	++	++	++	++
100	+	+	++	++
200	±	±	+	+
300	_	_	±	±
400	_	_	_	_
800	_	_	_	_

Where: (–): no growth (0%), (±): slight growth (25% -20%), (+): moderate growth (50% -30%), (++): good growth (90% - 70%), (+++): very good growth (100%
